# Infectious complications after CAR T-cell therapy: mechanisms, risk stratification, and prevention

**DOI:** 10.1007/s44313-026-00145-2

**Published:** 2026-06-11

**Authors:** Eunseuk Lee, Ji Hyun Lee, Hye Ryeon Kim, Seok Jae Huh, Suee Lee, Sung-Yong Oh, Sung-Hyun Kim, Hojin Lee

**Affiliations:** 1 Department of Internal Medicine, The State University of New Jersey, Rutgers, NJ USA; 2 Rutgers Health Community Medical Center, Toms River, NJ USA; 3 Division of Hematology-Oncology, Department of Internal Medicine, Dong-A University College of Medicine, 26, Daeshingongwon-Ro, Seo-Gu, Busan, 49201 Republic of Korea; 4 Division of Infectious Disease, Department of Internal Medicine, Dong-A University College of Medicine, Busan, Republic of Korea

**Keywords:** CAR T-cell therapy, Infection, Immunodeficiency, Hypogammaglobulinemia, Antimicrobial prophylaxis, Vaccination

## Abstract

Chimeric antigen receptor (CAR) T-cell therapy is an established treatment for relapsed or refractory B-cell malignancies. Despite therapeutic success, infectious complications remain a major contributor to non-relapsed morbidity and mortality. The risk of infection reflects a prolonged and multifactorial state of immunosuppression resulting from lymphodepleting chemotherapy, treatment-related cytopenia, sustained CD4^+^ T-cell lymphopenia, B-cell aplasia with hypogammaglobulinemia, and the use of corticosteroids or cytokine-directed therapies for the management of immune effector cell-associated toxicities (i.e., cytokine-release syndrome and immune effector cell–associated neurotoxicity). The spectrum of infections has evolved over time. Bacterial infections are observed most frequently in the early post-infusion period, whereas viral and opportunistic infections become more prominent later, particularly in patients with delayed immune recovery. Infection risk is also influenced by the underlying malignancy, prior exposure to treatment, and the type of CAR T-cell product used. For example, BCMA-directed CAR T-cell therapy has been associated with a higher infection burden, likely related to profound plasma cell depletion and sustained impairment of humoral immunity. Preventing infections in this setting requires a structured and individualized approach. Common strategies include antiviral and *Pneumocystis jirovecii* pneumonia prophylaxis, the selective use of antibacterial and antifungal prophylaxis during periods of severe or prolonged cytopenia, immunoglobulin replacement in patients with clinically significant hypogammaglobulinemia, and vaccination schedules guided by immune recovery. Therefore, coordinated and risk-adapted prevention is essential to minimize infectious complications and to improve long-term outcomes after CAR-T cell therapy. This review summarizes the current evidence and major recommendations of international guidelines to provide an updated overview of infection mechanisms, risk stratification, and preventive strategies for patients receiving CAR T-cell therapy.

## Introduction: CAR T-cell therapy and emerging infectious challenges

Chimeric antigen receptor (CAR) T-cell therapy has transformed the treatment landscape of hematological malignancies and is now widely used for several high-risk and relapsed diseases. It was first approved for patients with relapsed or refractory large B-cell lymphoma (LBCL) and B-cell acute lymphoblastic leukemia (B-ALL) and has since been expanded to include multiple myeloma (MM). In heavily pretreated patients, CAR T-cell therapy has substantially improved response and survival rates [1–7]. As indications continue to expand and eligibility criteria are broadened to include older and more comorbid patients, the global use of CAR T-cell therapy continues to grow [8–10].

With improved survival, non-relapse mortality (NRM) has emerged as a critical outcome [11, 12]. Although early attention was focused on disease control and acute toxicities, such as cytokine release syndrome (CRS) and immune effector cell–associated neurotoxicity syndrome (ICANS), longer follow-up has clarified that infections are a major contributor to NRM [11–13]. A recent meta-analysis reported that infections accounted for approximately half of non-relapse deaths after CAR T-cell therapy, exceeding mortality attributable to CRS, ICANS, and immune effector cell–associated hemophagocytic syndrome (IEC-HS) [11]. Treatment-related mortality in clinical trials generally ranges from 0 to 6%, whereas real-world NRM rates may reach 5% to 10%, highlighting infection as a key determinant of outcomes after CAR T-cell therapy [3, 11, 12]. Importantly, infectious complications occur across distinct temporal phases that reflect evolving immune dysfunction resulting from lymphodepletion, CAR T-cell activity, and immunosuppressive therapies [3, 14, 15].

This review provides an overview of infectious complications following CAR T-cell therapy, focusing on temporal risk patterns, underlying mechanisms, and evidence-based strategies for screening, antimicrobial prophylaxis, viral monitoring, immunoglobulin replacement, and vaccination [16, 17].

## Burden of infection after CAR T-cell therapy: incidence, timing and clinical impact

Infectious complications are common after CAR T-cell therapy and remain important contributors to morbidity and NRM. Across trials and real-world cohorts, reported infection rates range from approximately 15% to 50%, reflecting differences in patient populations, CAR T-cell constructs, follow-up duration, and supportive care practices [14, 15, 18, 19]. Infection-related mortality accounts for approximately 2–4% of deaths and is a significant component of NRM [11, 18, 19]. NRM also varies according to disease type, with higher rates reported in mantle cell lymphoma (10.6%) and MM (8.0%) than in LBCL (6.1%) or indolent lymphoma (5.7%) [11].

The infection risk is time-dependent and closely linked to the evolving immune dysfunction that follows CAR T-cell therapy. Three clinical phases are commonly described: early (< 30 days), prolonged (30–90 days), and late (> 90 days) [14–16]. This temporal framework can help guide strategies for infection surveillance, antimicrobial prophylaxis, and vaccination.

During the early phase (days 0–30), patients experienced a hematologic nadir following lymphodepleting chemotherapy and CAR T-cell infusion. Profound neutropenia and lymphopenia are nearly universal and represent the major drivers of the risk of early infection [3, 16]. Neutrophil recovery typically begins within 2–4 weeks, although recovery kinetics vary widely [20–22]. Some patients demonstrate a prompt and sustained recovery, whereas others develop delayed or biphasic cytopenia characterized by secondary decline after initial improvement [23]. These patterns may reflect bone marrow suppression, inflammatory signaling associated with CAR T-cell expansion, or limited marrow reserves from previous therapies [20, 21, 24]. Prolonged neutropenia is strongly associated with an increased risk of bacterial and invasive fungal infections (IFI) [14, 16].

Clinically, bacterial infections predominate in the early phases and are the leading cause of infectious morbidity [3, 15]. Gram-negative bacteremia, including *Escherichia coli* and *Pseudomonas aeruginosa*, as well as gram-positive infections, such as *Staphylococcus* and *Enterococcus* species, are commonly reported. These infections are often present as bloodstream pneumonia or catheter-associated infections [19]. Frequent exposure to antibiotics and gastrointestinal toxicity also increase the probability of *Clostridioides difficile (C. difficile)* infection, which should be considered in patients with similar symptoms. Early fungal infections are less common but may occur in patients with prolonged neutropenia or prior extensive immunosuppression, with *Candida* species being the most frequently reported [3, 25]. Viral infections are less prominent in the early phase but may emerge in patients who require high-dose corticosteroids or cytokine-directed therapy for severe CRS or ICANS [26, 27]. Reactivation of cytomegalovirus (CMV) infection has been described in selected high-risk populations within the first two to three weeks of infusion [26].

Several clinical factors contribute to delayed hematologic recovery and increased risk of infection after CAR T-cell therapy. High disease burden at infusion, extensive exposure to prior chemotherapy, previous autologous or allogeneic hematopoietic stem cell transplantation (HSCT), and baseline cytopenia are consistently associated with a diminished bone marrow reserve [20, 22, 28]. Severe CRS has also been associated with prolonged cytopenias, likely mediated by cytokine-driven marrow suppression and endothelial activation [20, 29]. Additionally, elevated levels of inflammatory markers such as ferritin and CRP further support the idea that systemic inflammation contributes to bone marrow dysfunction and delayed hematologic recovery after CAR T-cell therapy [21]. Persistent anemia and thrombocytopenia are often accompanied by neutropenia, which contributes to transfusion dependence, recurrent healthcare encounters, and cumulative immunological stress.

During the prolonged phase (days 30–90), neutrophil counts frequently recover but adaptive immune dysfunction persists. B-cell aplasia, hypogammaglobulinemia, and delayed CD4^+^ T-cell reconstitution continue to impair host defenses [3, 30, 31]. Consequently, viral and opportunistic infections have become increasingly prevalent [15, 16]. Respiratory viral infections, including influenza, respiratory syncytial virus, parainfluenza, and SARS-CoV-2, are the main sources of morbidity during this period and may require hospitalization [3, 15]. Herpes simplex virus (HSV) and varicella-zoster virus (VZV) infections can occur in the absence of antiviral prophylaxis [27]. CMV infections may persist or develop during this phase, particularly in patients receiving prolonged immunosuppressive therapy [26].

Fungal infections during the prolonged phase are less frequent than bacterial infections seen earlier after infusion, but cause substantial mortality when they occur [25]. Invasive mold infections, including *Aspergillus* species, are most commonly observed in patients with prolonged cytopenia, prior allogeneic hematopoietic cell transplantation, or exposure to high-dose corticosteroids [3, 25]. *Pneumocystis jirovecii* (PJP) has also emerged as a clinically relevant opportunistic infection during this window, particularly in patients who have not received adequate prophylaxis or with delayed T-cell recovery [16, 32].

Beyond 90 days after CAR T-cell therapy, the infection risk decreases but does not resolve completely [14, 30]. Late infections are largely driven by persistent humoral and cellular immune dysfunction rather than cytopenia [30, 33]. Persistent B-cell aplasia and hypogammaglobulinemia predispose patients to recurrent sinopulmonary infections, often caused by encapsulated bacteria [17, 30]. These infections may be repetitive and tend to contribute more to chronic morbidity than to acute mortality [30]. Viral susceptibility also persists in the late phase with an ongoing risk of respiratory viral infections and herpes virus reactivation [3, 30]. Vaccine responses are frequently suboptimal in this population, further complicating infection prevention efforts [30]. Opportunistic infections such as PJP or invasive fungal disease are rare, but may occur in patients with sustained immunosuppression or delayed immune reconstitution [30].

**Fig. 1:**
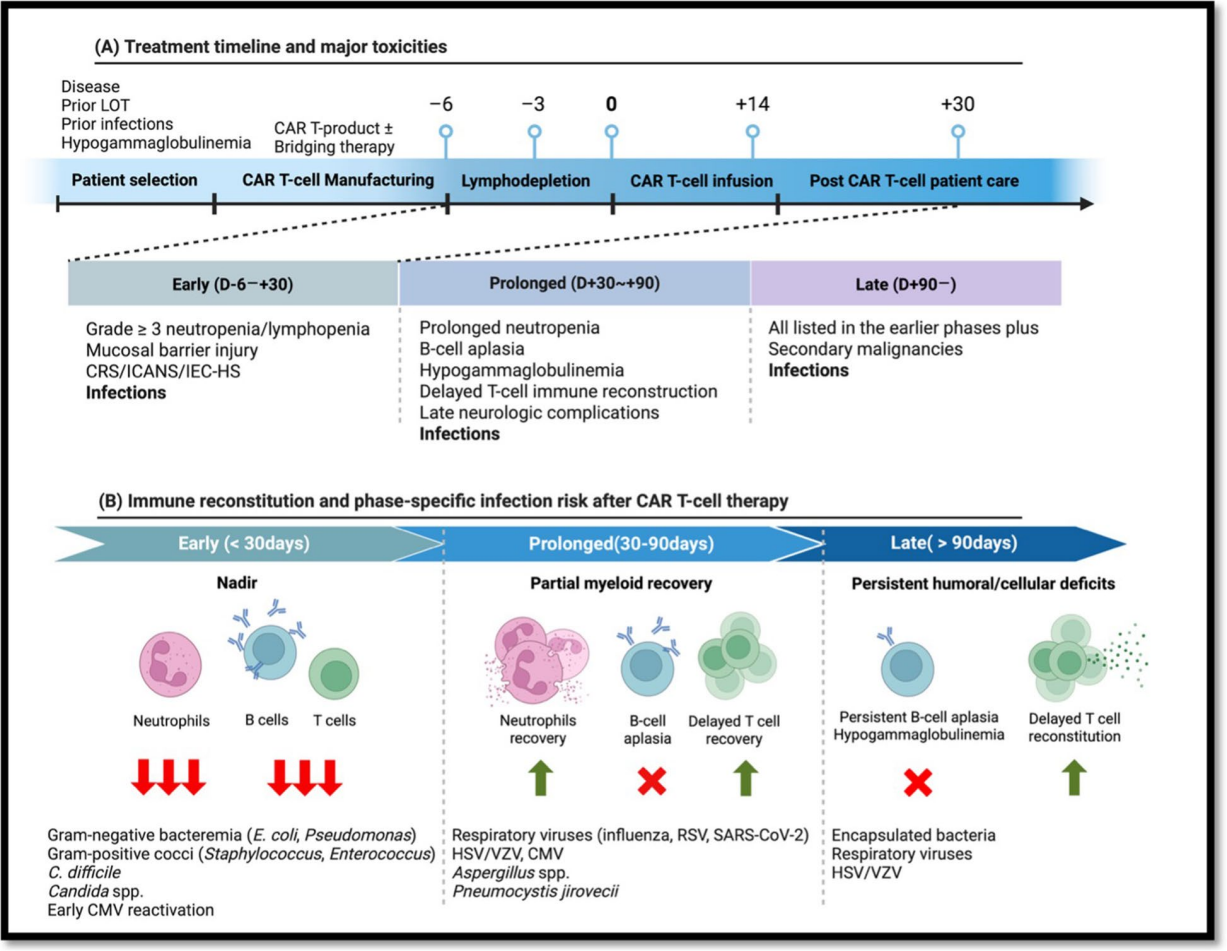
Temporal evolution of toxicities, immune reconstitution, and infection risk after CAR T-cell therapy. **A** Schematic overview of the CAR T cell therapy timeline and clinical factors that may influence infection risk during each treatment phase. The treatment course included patient selection, CAR T-cell manufacturing (with or without bridging therapy), lymphodepleting chemotherapy, CAR T-cell infusion, and post-infusion care. Clinical variables, such as prior lines of therapy, prior infections, baseline hypogammaglobulinemia, and treatment-related toxicities, may contribute to infection susceptibility during the early, prolonged, and late phases after CAR T-cell therapy. The early phase (day − 6 to + 30) is characterized by grade ≥ 3 neutropenia/lymphopenia, CRS, ICANS, and other immune effector cell-associated toxicities. The prolonged phase (days + 30 to + 90) included persistent cytopenia, B-cell aplasia, hypogammaglobulinemia, delayed T-cell reconstitution, and delayed neurotoxicity. The late phase (> 90 days) includes sustained humoral deficits and secondary malignancies in addition to the complications listed in the earlier phases. Infectious complications can occur throughout the course of CAR T-cell therapy. **B** Immune reconstitution and phase-specific infection risk after CAR T cell therapy. Patients experience a hematologic nadir with profound neutropenia and lymphopenia during the early phase (< 30 days). In the prolonged phase (30–90 days). Neutrophil recovery occurs, B cell aplasia persists, and CD4 + T cell recovery remains delayed. In the late phase (> 90 days), persistent B-cell aplasia and hypogammaglobulinemia persist despite gradual T-cell recovery, contributing to ongoing susceptibility to bacterial, viral, and opportunistic infections. Red downward arrows indicate depletion, green upward arrows indicate recovery, and the red X symbols indicate persistent absence or impairment. Abbreviations: CAR, Chimeric antigen receptor; LOT, lines of therapy; CRS, cytokine release syndrome; ICANS, immune effector cell-associated neurotoxicity syndrome; IEC-HS, immune effector cell-associated hemophagocytic syndrome; CMV, cytomegalovirus; RSV, respiratory syncytial virus; HSV, herpes simplex virus; VZV, varicella zoster virus. *Created with BioRender.com*

Together, these temporal patterns highlight that the risk of infection after CAR T-cell therapy is dynamic and closely related to the pace of immune recovery [14, 15]. Therefore, a time-adapted framework for infection surveillance and prophylaxis is essential to minimize NRM and improve long-term outcomes in CAR T-cell recipients [14]. These phases, their associated immune deficits, and infection risks are summarized in Fig. 1.

## Pathophysiology of infection risk after CAR T-cell therapy

### Immune dysregulation

Susceptibility to infection after CAR T cell therapy reflects dynamic immune dysregulation rather than a single immunological defect. Lymphodepleting chemotherapy, CAR T-cell–mediated cytotoxicity, and immunosuppressive therapies used to manage therapy-related toxicities collectively shape the timing and spectrum of infections [3, 14, 15].

Early risk is primarily driven by profound cytopenia induced by lymphodepleting regimens. Nearly all patients develop neutropenia and lymphopenia, which alter innate and adaptive immune responses [14, 15]. Delayed or biphasic cytopenia may extend the vulnerability to the prolonged phase.

Delayed CD4^+^ T-cell reconstitution, including prolonged CD4^+^ lymphopenia and functional impairment, further increases susceptibility to viral and opportunistic infections, particularly beyond the early post-infusion phase [15, 34].

Immunosuppressive management of complications associated with immune effector cells further increases this risk. Corticosteroids and cytokine-targeted therapies (i.e., tocilizumab, anakinra) impair innate and adaptive immune function in a dose-dependent manner [3, 26, 33]. Patients requiring prolonged or multi-agent immunosuppression represent a particularly high-risk subgroup [11, 26, 32, 33].

Finally, host-related factors such as older age, prior therapy burden, prior HSCT, and comorbidities interact with CAR-T cell-specific immune perturbations to shape individual infection risks [14, 15, 19] (Table 1).

**Table 1:** Summary of infection risk factors after CAR T-cell therapy

Category	Risk factor	Impact on infection risk
**Treatment-related**	Lymphodepleting chemotherapy	Early bacterial and fungal infections
	Grade ≥ 3 CRS	Increased risk of CMV reactivation and prolonged cytopenias
	Prolonged or high-dose corticosteroids	Independent predictor of bacterial, viral, and fungal infections
	Use of ≥ 2 immunosuppressive agents (e.g., tocilizumab + anakinra)	Opportunistic infections, CMV reactivation
**Hematologic**	Prolonged or biphasic neutropenia	~ 60–80% in early phase, bacterial and invasive fungal infections
	Delayed CD4 recovery (< 200/µL)	Viral infections, *Pneumocystis jirovecii* pneumonia
**CAR T construct**	CD19-directed CAR T	B-cell aplasia, hypogammaglobulinemia
	BCMA-directed CAR T	Profound plasma cell depletion, severe humoral immunodeficiency
**Patient-related**	Older age, extensive prior therapy, prior HSCT	Delayed immune recovery, higher NRM
**Baseline immunity**	Hypogammaglobulinemia, CMV seropositivity	Recurrent infections, CMV reactivation (variable incidence in seropositive patients; higher with immunosuppression)

The risk factors shown represent commonly reported clinical variables associated with increased infection risk following CAR T-cell therapy, based on observational studies and registry analyses. Multiple factors frequently coexist in individual patients and may have additive effects on the susceptibility to infection

*Abbreviations: CAR* chimeric antigen receptor T-cell therapy, *CRS* cytokine release syndrome, *CMV* cytomegalovirus, *HSCT* hematopoietic stem cell transplantation, *NRM* nonrelapse mortality

### Differences in the risk of infection based on CAR T-cell construct and disease entity

The risk of infection after CAR T-cell therapy is not uniform across all products or disease settings. Increasing clinical experience has highlighted meaningful differences in both the incidence and nature of infections based on the CAR T-cell construct and the underlying malignancy being treated [14, 34]. Among these, recipients of BCMA-directed CAR T-cell therapy appear to carry a particularly high infection burden compared with those treated with CD19-directed CAR T-cell products [11, 34, 35].

Lineage-specific immune defects contribute to susceptibility to infections. CD19-directed CAR T-cell therapy induces prolonged B-cell aplasia, leading to hypogammaglobulinemia and impaired humoral immunity [3, 30, 31, 36]. In contrast, BCMA-directed CAR T-cell therapy directly depletes plasma cells, resulting in a more profound and sustained antibody deficiency [30]. A central mechanistic driver of the increased risk of infection with BCMA-directed CAR T cell therapy is profound humoral immunodeficiency. In plasma cell–driven diseases, such as MM, this effect is further compounded by pre-existing immune dysfunction and cumulative prior therapies, resulting in severe and prolonged hypogammaglobulinemia [37–40]. Comparative studies suggest that although early bacterial infection rates are broadly similar between CD19- and BCMA-directed CAR T-cell recipients, the pattern of infections diverges over time. In BCMA-directed CAR T cell therapy, subsequent infections, particularly respiratory viral and recurrent sinopulmonary bacterial infections, are more frequent and persistent. Opportunistic infections, including PJP and IFI, are also more frequently reported, particularly in patients who require prolonged corticosteroid exposure or additional immunosuppressive therapies [14, 16, 32, 34, 35].

In contrast, CD19-directed CAR T-cell therapy is more commonly associated with B-cell aplasia than with complete plasma cell depletion [3, 41]. Although hypogammaglobulinemia is still frequent, immunoglobulin levels may recover over time, and the infection risk may decline accordingly [14, 41]. Nevertheless, patients with delayed B-cell recovery remain vulnerable to late infections, reflecting the heterogeneity within CD19-directed treatment populations [17, 41].

In addition to the target antigen–related differences, the costimulatory domain may further modulate the risk of infection. CD28-based CAR T-cell constructs are associated with rapid expansion and higher rates of severe CRS and ICANS, often requiring immunosuppressive therapy and contributing to an increased risk of early infection. In contrast, 4-1BB-based constructs exhibit prolonged persistence, which can lead to sustained B-cell aplasia and a greater burden of late infections [42–45].

Disease-specific factors further increase the risk of infection in patients with MM. Patients with myeloma often enter CAR T-cell therapy with advanced age, extensive prior exposure to immunosuppressive agents, baseline cytopenia, renal dysfunction, and compromised marrow reserve. The prior use of proteasome inhibitors, immunomodulatory drugs, monoclonal antibodies, and corticosteroids contributes to cumulative immune exhaustion [32, 39]. In addition, myeloma-related immune paresis and impaired antigen presentation persist even after disease response, limiting immune recovery despite effective tumor control [39, 40].

**Fig. 2:**
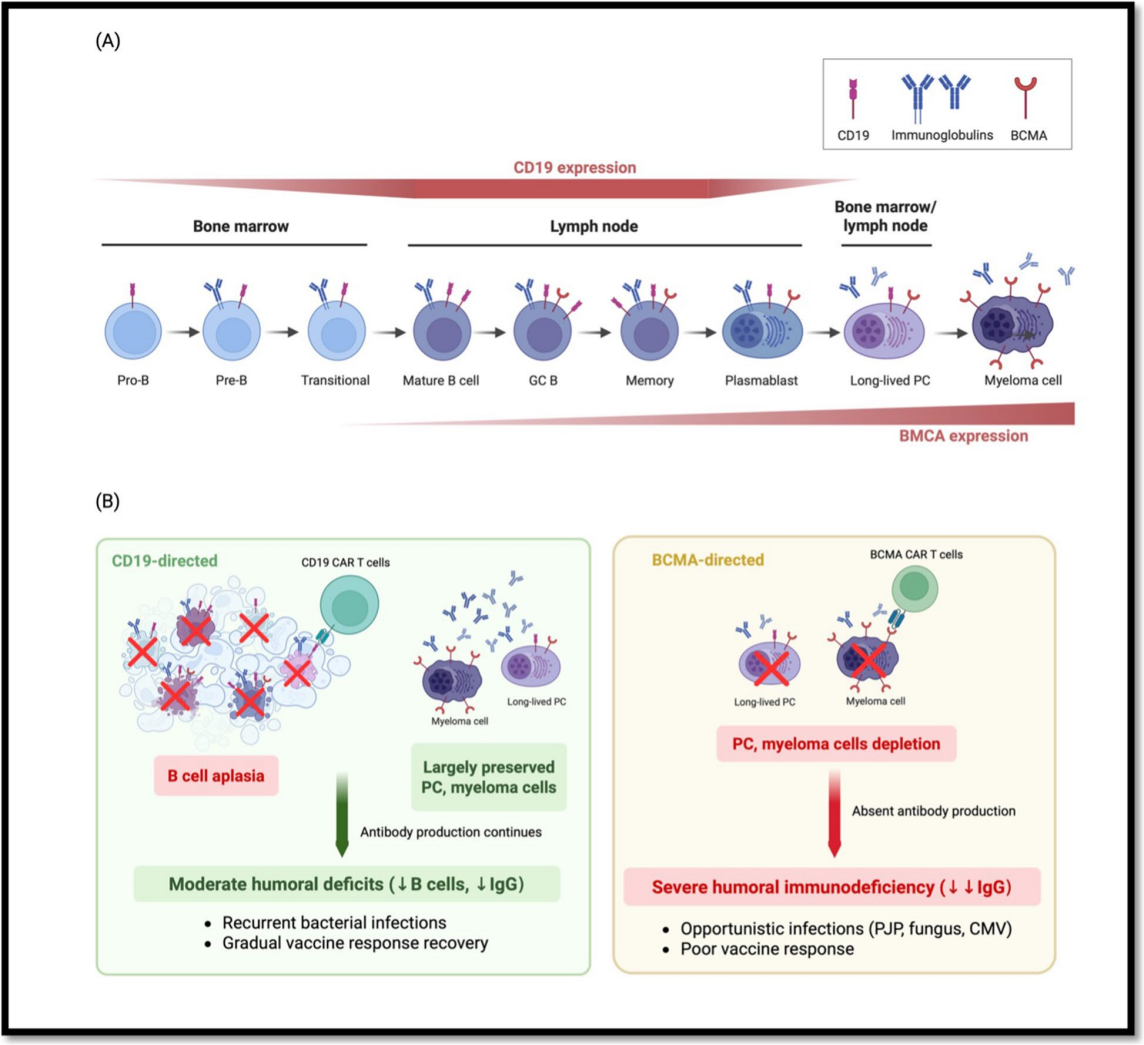
Differential humoral immune depletion following CD19- versus BCMA-directed CAR T-cell therapy. **A** Expression of CD19 and BCMA across the B cell differentiation pathway in the bone marrow and lymph nodes. CD19 is expressed from early B-cell precursors through mature and memory B cells but is absent in terminally differentiated long-lived PCs. In contrast, BCMA expression was enriched in plasmablasts, long-lived PCs, and myeloma cells, with minimal expression in the earlier B-cell stages. **B** Functional consequences of lineage-specific targeting. CD19-directed CAR-T cell therapy induces B cell aplasia through the depletion of CD19^+^ B cells; however, long-lived PCs may remain relatively preserved, allowing partial maintenance of antibody production and resulting in moderate reduction in immunoglobulin levels. In contrast, BCMA-directed CAR T-cell therapy directly depletes BCMA^+^ Plasma cells, including long-lived antibody-producing cells, within the bone marrow niche. This results in profound hypogammaglobulinemia, impaired pathogen-specific immunity, and an increased susceptibility to recurrent and opportunistic infections. Abbreviations: Pro B, pro-B cell; Pre B, pre-B cell; GC B, germinal center B cell; PC, plasma cell; BCMA, B-cell maturation antigen; PJP, *Pneumocystis jirovecii* pneumoniae; CMV, cytomegalovirus. *Created with BioRender.com*

These overlapping factors help explain why infection-related morbidity and mortality appear disproportionately higher in patients with MM undergoing CAR T-cell therapy [11, 35]. Our findings have several practical implications. They support earlier considerations of immunoglobulin replacement, heightened vigilance for viral infections, and more aggressive risk-adapted prophylactic strategies in BCMA-directed CAR-T cell recipients [17, 32]. As newer CAR T-cell constructs and bispecific therapies continue to emerge, understanding construct- and disease-specific infection risks is essential to refine prevention strategies and minimize NRM [46, 47]. The mechanistic differences in humoral immune depletion with CD19- versus BCMA-directed CAR-T cell therapy are illustrated in Fig. 2.

A similar pattern of disease-related vulnerability has been observed in other lymphoma subtypes, particularly mantle cell lymphoma (MCL). Patients with MCL receiving CAR T-cell therapy often represent an older and heavily pretreated population with limited bone marrow reserve and baseline immune dysfunction. Although product-related factors, including the toxicity profile of brexucabtagene autoleucel, may contribute to the higher NRM observed in the MCL group, it is more likely driven by cumulative patient vulnerability and infection-related complications, which ultimately translate into an increased burden of severe and sometimes fatal infections [48].

### Hematologic recovery and infection susceptibility

Hematologic recovery after CAR T-cell therapy is heterogeneous and plays a central role in determining susceptibility to infection across all post-infusion phases. Although early cytopenias are nearly universal and largely attributable to lymphodepleting chemotherapy, a substantial proportion of patients experience delayed, prolonged, or biphasic cytopenias that extend vulnerability to infection beyond the first month after treatment [21, 22, 49].

Neutrophil recovery following CAR T-cell infusion typically begins within 2–4 weeks; however, recovery kinetics vary widely [22, 49]. Some patients demonstrate rapid and sustained neutrophil recovery, whereas others develop delayed recovery or secondary decline after the initial improvement [50]. These biphasic patterns are increasingly recognized and may reflect inflammatory signaling associated with CAR T-cell expansion or limited bone marrow reserve from prior therapies [21, 24, 49]. In patients with prolonged or unexplained cytopenia, a bone marrow evaluation may be required to assess the marrow reserve and exclude alternative etiologies [49]. Importantly, prolonged neutropenia is strongly associated with an increased risk of bacterial and IFI [14, 16].

Several clinical factors have been associated with delayed or prolonged cytopenia after CAR T-cell therapy, including a high disease burden at infusion, extensive prior chemotherapy exposure, prior autologous or allogeneic HSCT, and baseline cytopenia [21, 28]. Severe CRS has also been associated with prolonged cytopenias, likely mediated through cytokine-driven marrow suppression and endothelial activation [29, 49]. Elevated levels of inflammatory markers such as ferritin and CRP are similarly associated with delayed hematologic recovery [22].

These observations have led to the development of risk stratification tools to better predict hematological toxicity after CAR T-cell therapy. One such model, the CAR-HEMATOTOX score, integrates baseline hematological and inflammatory parameters to estimate the risk of immune effector cell–associated hematotoxicity. Higher CAR-Hematotox scores are associated with prolonged cytopenia, severe infections, and increased NRM. Prospective validation studies have further supported its ability to identify patients at high risk of delayed hematologic recovery and infection-related complications [32, 34, 51].

The clinical consequences of prolonged cytopenia extend beyond those of neutropenia. Persistent anemia and thrombocytopenia contribute to transfusion dependence and increased healthcare utilization, whereas recurrent hospitalization and prolonged antimicrobial exposure may further increase the risk of resistant infections. These factors may collectively increase the risk of infection during the prolonged and late post-infusion phases [22, 49].

Granulocyte colony-stimulating factor (G-CSF) is frequently used to support neutrophil recovery after CAR-T cell therapy, although its optimal timing remains debatable. Early concerns suggested that G-CSF may exacerbate CRS or ICANS by enhancing myeloid activation. However, emerging clinical experience suggests that delayed administration, typically after resolution of CRS and ICANS, does not significantly increase toxicity and may shorten the duration of neutropenia [50, 52]. Consequently, many centers avoid G-CSF during the first 5 days after infusion and initiate G-CSF treatment approximately 7–14 days (or later) in patients with persistent grade 3–4 neutropenia and high infection risk [17, 28, 53].

Despite evolving experience, no universally accepted strategy has been proposed for managing post–CAR T-cell cytopenia. Therefore, clinical decisions regarding the use of growth factors, transfusion support, and infection prophylaxis are often individualized, based on patient-specific risk factors and institutional practices [33, 49]Understanding cytopenia kinetics and marrow reserve provides essential context to interpret risk of infection across post-infusion phases and informs subsequent discussions on targeted prophylaxis and monitoring strategies [21, 22].

## Antimicrobial prophylaxis and vaccination: evidence-based and risk-adapted prevention

Antimicrobial prophylaxis is a central component of infection prevention after CAR T-cell therapy, but must balance efficacy with toxicity, antimicrobial resistance, and risk of overtreatment [16, 33]. Current preventive strategies are largely extrapolated from HSCT and intensive chemotherapy settings, although specific evidence relative to CAR cells and guideline recommendations are emerging [54, 55]. Prevention of infection after CAR T-cell therapy generally follows a phase-adapted and risk-stratified approach. Key international guideline recommendations for antibacterial, antiviral, and antifungal prophylaxis from the European Society for Blood and Marrow Transplantation (EBMT) and the American Society for Transplantation and Cellular Therapy (ASTCT) are summarized and compared in Table 2. Practical antimicrobial prophylaxis and vaccination strategies after CAR T-cell therapy are summarized in Table 3.

**Table 2:** Summary of guideline recommendations from the european society for blood and marrow transplantation (EBMT) and the american society for transplantation and cellular therapy (ASTCT) for antimicrobial prophylaxis after CAR T-cell therapy

**Domain**	**EBMT **[56]	**ASTCT **[57]
Antibacterial prophylaxis	**Consider during neutropenia**, extrapolated from HSCT practice; institutional resistance patterns should be considered	**Selective approach**; fluoroquinolone prophylaxis reasonable during neutropenia; close monitoring acceptable
Antifungal prophylaxis (yeast)	Fluconazole during neutropenia	Fluconazole during neutropenia
Antifungal prophylaxis (mold)	**Not routinely recommended**; consider in high-risk patients only with defined risk factors	**High-risk patients only with** defined risk factors
HSV/VZV prophylaxis	**Universal prophylaxis;** extrapolated from HSCT practice	**Universal prophylaxis;** starting with lymphodepletion; prolonged duration
CMV prophylaxis	**Not recommended** due to insufficient evidence	**Not recommended**
PJP prophylaxis	**Recommended for all patients**; ≥ 6 months; duration guided by immune recovery	** ≥ 6 months; continuation often guided by** CD4 < 200 cells/µL
HBV reactivation prevention	**Mandatory antiviral prophylaxis** for HBsAg +; prophylaxis or close DNA monitoring for anti-HBcAg +	**Mandatory prophylaxis for HBsAg +; prophylaxis or surveillance for anti-HBcAg + **
IVIG replacement	**Selective use** in hypogammaglobulinemia with recurrent or severe infections; routine prophylaxis not endorsed	**Selective use when** IgG ≤ 400 mg/dL with infections; lower threshold in pediatric patients
Vaccination	**Delayed revaccination;** extrapolated from HSCT; start ≥ 3–6 months post-CAR-T depending on immune recovery	**Staged revaccination**: influenza/COVID-19 ~ 3 months; other inactivated ≥ 6 months; live ≥ 12 months with immune recovery

Recommendations were adapted from the current EBMT and ASTCT consensus guidelines. Specific prophylactic strategies may vary according to the institutional practices, patient risk factors, and local epidemiology

*Abbreviations: EBMT* European Society for Blood and Marrow Transplantation, *ASTCT* American Society for Transplantation and Cellular Therapy, *HSCT* herpes simplex virus, *HSV* hematopoietic stem cell transplantation, *VZV*, varicella zoster virus, *CMV* cytomegalovirus, *PJP Pneumocystis jirovecii* pneumonia, *HBV* hepatitis B virus, *HBsAg* hepatitis B surface antigen, *anti-HBc*, antibody to hepatitis B core antigen, *IVIG* intravenous immunoglobulin

**Table 3:** Practical antimicrobial prophylaxis and vaccination strategies after CAR T-cell therapy based on current guideline recommendations

Type of prophylaxis	Indication	Recommended strategy	Duration
Antimicrobial prophylaxis			
** Antibacterial**	ANC < 500/µL	Fluoroquinolone (if local resistance acceptable)	Until neutrophil recovery
** Antifungal (yeast)**	Neutropenia	Fluconazole	Until neutrophil recovery
** Antifungal (mold)**	Prolonged neutropenia, prior IFI, prior allo-HCT, high-dose steroids, CRS/ICANS escalation	Posaconazole, isavuconazole, or echinocandin (avoid voriconazole with ICANS)	Through risk period; ≥ 1 month after steroid cessation
** HSV/VZV**	All CAR T recipients	Acyclovir or valacyclovir	≥ 1 year; often extended
** PJP**	All CAR T recipients	TMP-SMX (alternatives if intolerant)	≥ 6 months and CD4 > 200/µL
** CMV**	Only High-risk patients	PCR surveillance (no routine prophylaxis)	2–6 weeks post-infusion or up to 30 days after steroid cessation
Vaccination			
** Inactivated influenza vaccine**	All CAR T recipients	Annual inactivated influenza vaccine	Start ~ 3 months after CAR T; annually
** COVID-19 vaccine**	All CAR T recipients	Updated COVID-19 vaccination per current schedule	Start ~ 3 months after CAR T
** Pneumococcal vaccine**	All CAR T recipients	Conjugate pneumococcal vaccine series	Start ~ 6 months after CAR T
** Routine inactivated vaccines**	Patients with immune recovery	DTaP/Tdap, Hib, hepatitis A/B, IPV, HPV, RSV	Start ~ 6 months after CAR T
** Live vaccines**	Selected patients with immune reconstitution	MMR, VZV or other live vaccines, if appropriate	≥ 24 months after CAR T

The table summarizes practical infection prevention strategies based on current international guidelines, including recommendations from the EBMT [56], ASTCT [57], and NCCN [16]. Specific antimicrobial regimens and vaccination schedules may be adapted according to local epidemiology, institutional protocols, and patient-specific risk factors

*Abbreviations: ANC* absolute neutrophil count, *IFI* invasive fungal infection, *allo-HCT* allogeneic hematopoietic cell transplantation, *CRS* cytokine release syndrome, *ICANS* immune effector cell–associated neurotoxicity syndrome, *HSV* herpes simplex virus, *VZV* varicella-zoster virus, *PJP Pneumocystis jirovecii* pneumonia, *TMP-SMX* trimethoprim–sulfamethoxazole, *CMV* cytomegalovirus, *CAR T* chimeric antigen receptor T-cell, *DTaP/Tdap* diphtheria-tetanus-acellular pertussis vaccine, *Hib Haemophilus influenzae* type b vaccine, *IPV* inactivated poliovirus vaccine, *HPV* human papillomavirus, *RSV* respiratory syncytial virus, *MMR* measles-mumps-rubella

### Antibacterial prophylaxis

Fluoroquinolone prophylaxis is selective, rather than universal. The National Comprehensive Cancer Network (NCCN) and the American Society of Clinical Oncology (ASCO)/Infectious Diseases Society of America (IDSA) recommend fluoroquinolone prophylaxis use during severe neutropenia, considering institutional resistance patterns and *Clostridioides difficile* infection [58]. In CAR T-cell recipients, antibacterial prophylaxis is typically reserved for patients with an absolute neutrophil count (ANC) < 500/μL and is discontinued upon neutrophil recovery [16, 54, 59]. Empiric broad-spectrum antimicrobial therapy should not be delayed in febrile patients even when CRS is suspected [22].

### Antiviral prophylaxis

HSV and VZV prophylaxis with acyclovir or valacyclovir is universally recommended, typically initiated with lymphodepleting chemotherapy and continued for at least 1 year [32, 54]. This recommendation reflects prolonged T-cell dysfunction beyond the neutropenic period [16, 27]. Routine prophylaxis for cytomegalovirus (CMV) is not recommended; instead, a risk-adapted surveillance approach is generally employed in selected high-risk patients. Further details regarding CMV reactivation patterns and management are discussed in Sect. "CMV Reactivation" [26, 60–63].

### Antifungal prophylaxis

Fluconazole is commonly used to prevent candidiasis [16, 32]. Mold-active prophylaxis is generally reserved for patients with established risk factors, including prolonged neutropenia, prior invasive fungal infection (IFI), prior allo-HSCT, or prolonged or high-dose corticosteroid exposure [16, 51, 64].

In addition to the EBMT and ASTCT guidelines, the updated ECIL-10 recommendations provide a more structured, risk-adapted framework for antifungal prophylaxis in CAR-T cell recipients. Notably, ECIL-10 distinguishes between pre-infusion factors (e.g., prior therapies, baseline cytopenia, and prior IFI) and post-infusion factors (e.g., CRS requiring corticosteroids, prolonged neutropenia, or the use of additional immunosuppressive agents) and suggests that mold-active prophylaxis should be considered when such risk factors are present. This approach extends previous guideline recommendations by incorporating dynamic treatment-related risk factors specific to CAR T-cell therapy [51].

### Vaccination after CAR T-cell therapy

Current guidelines and expert consensus statements suggest that vaccination after CAR T cell therapy should be considered in the context of delayed and variable immune reconstitution. Inactivated vaccines are generally considered safe and are typically initiated approximately 3–6 months after infusion, whereas live vaccines should be deferred following more complete immune recovery, often 12–24 months post-therapy [65–70].

Vaccine responsiveness is frequently reduced after CAR T-cell therapy, largely reflecting impaired humoral immunity [67, 71, 72]. Although responses may improve with B-cell recovery, serological measures alone do not consistently predict protective immunity [73]. Vaccination is generally used as a complementary strategy along with antimicrobial prophylaxis and intravenous immunoglobulin (IVIG) in high-risk patients [65, 66]. Vaccination of household contacts is also commonly recommended to improve indirect protection [16, 65, 74]. Given the limited prospective data in this setting, current practice often relies on a pragmatic, time-based approach with selective consideration of immune recovery and the clinical context [65, 67].

## Role of intravenous immunoglobulin

Hypogammaglobulinemia is a common complication after CD19- and BCMA-directed CAR T-cell therapy and contributes to intermediate and late risk of infection [17, 75]. IVIG provides passive antibody replacement and may enhance opsonization and immune modulation [75, 76]. Current practice favors selective administration [17, 75]. Most guidelines recommend IVIG in patients with serum IgG levels < 400–600 mg/dL accompanied by recurrent or severe infections. The typical dose is 400–500 mg/kg of IVIG every 3–4 weeks, with the practical goal of maintaining serum IgG at > 400–600 mg/dL. BCMA-directed CAR T-cell therapy, particularly for patients with MM, may warrant earlier consideration because of plasma cell depletion and sustained antibody deficiency [17, 32, 40]. Routine prophylactic IVIG is not recommended for any recipients (Table 4).

**Table 4:** Indications for IVIG After CAR T-cell therapy

CAR T construct	Suggested IVIG indication	Rationale
CD19-directed	IgG < 400–600 mg/dL **with recurrent or severe infections**	B-cell aplasia with variable recovery [16, 17, 75]
BCMA-directed (MM)	IgG < 400 mg/dL (even without documented infection)	Profound plasma cell depletion and sustained humoral deficiency [17, 32, 77]
Pediatric patients	Lower threshold for initiation	Higher infection susceptibility [74]
Discontinuation	IgG normalization and infection resolution	Avoid overtreatment [17, 75]

The suggested thresholds reflect commonly used clinical practice patterns reported in the literature and expert guidance. Decisions regarding IVIG initiation and discontinuation should consider the infection history, degree of hypogammaglobulinemia, and recovery of humoral immunity

*Abbreviations: CAR T* chimeric antigen receptor T-cell therapy, *IVIG* intravenous immunoglobulin, *IgG* immunoglobulin G, *MM* multiple myeloma

IVIG use must balance benefits with cost and infusion burden and should be guided by the clinical context, including infection history and degree of immune recovery, while considering potential adverse effects, such as thrombosis and renal dysfunction [75–77].

## Infection screening and monitoring

### Baseline screening

Pre–CAR T evaluation includes HIV testing, hepatitis B and C serologies, and evaluation of CMV status, with antiviral prophylaxis for patients at risk of hepatitis B reactivation [16, 17, 78, 79]. Screening for tuberculosis, *Strongyloides*, or endemic fungi infections should be guided by epidemiologic exposure risk [54, 80]. Active infections should be identified and controlled before administering lymphodepleting chemotherapy.

### Post–CAR T surveillance

Routine surveillance culture is not recommended for asymptomatic patients. Instead, post-CAR T cell monitoring is guided by clinical status and individual risk factors [32]. CMV polymerase chain reaction (PCR) monitoring is generally performed in high-risk patients. A detailed discussion of CMV reactivation patterns, risk factors, and management strategies is provided in Sect. "CMV Reactivation" [26, 32].

### Distinguishing infection from CRS/ICANS

Fever, hypotension, and neurological symptoms overlap between infections and immune effector cell-associated toxicity [3, 16, 17]. Therefore, clinicians should maintain a low threshold for diagnostic evaluation and empirical antimicrobial therapy when an infection is suspected. Biomarkers such as CRP and ferritin are nonspecific and should be interpreted with caution in this setting [17, 33]. Interleukin-6 (IL-6) levels are also of limited diagnostic value, particularly in patients who have received anti-IL-6 therapy, as circulating IL-6 levels may remain elevated due to receptor blockade. In patients with persistent fever after CAR T-cell infusion, careful evaluation of atypical infections, including viral, fungal, and opportunistic pathogens, is essential. At the same time, clinicians should consider the possibility of IEC-HS and perform appropriate laboratory assessments, including liver function tests and fibrinogen levels, to allow timely recognition and management, including anti-IL-1–directed therapy when indicated.

Therefore, infection screening and monitoring after CAR T-cell therapy requires careful baseline evaluation and risk-adapted surveillance during follow-up [36, 80]. Early recognition of infection is essential for guiding diagnostic evaluation and timely antimicrobial therapy in this highly immunocompromised population.

## Special clinical scenarios

### COVID-19

CAR T recipients may experience prolonged viral shedding and impaired vaccine responses due to B cell depletion [81–84]. Infection that occurs early after infusion or during periods of immunosuppression carries an increased risk of severe disease [60, 81]. Thus, early antiviral therapy and repeated diagnostic testing may be required in patients with persistent symptoms [61, 84].

The management of SARS-CoV-2 infection before CAR T-cell therapy should be guided by symptom severity. In symptomatic patients, CAR T-cell infusion is generally delayed for approximately 7–14 days until clinical improvement is achieved. Repeated SARS-CoV-2 testing after symptom resolution is not always required. Antiviral therapies, such as remdesivir or nirmatrelvir/ritonavir, should be considered when clinically indicated.

In patients with asymptomatic or mild infections, CAR T-cell therapy may be considered in selected cases, although delayed treatment is generally preferred when feasible. Clinical decision-making should consider symptom severity, evidence of lower respiratory tract involvement, inflammatory markers, and the urgency of treating the underlying malignancy [62].

### Invasive fungal infections

Although IFI is relatively rare after CAR T-cell therapy, it is associated with substantial mortality and occurs in a setting of cumulative and evolving immunosuppression. This risk reflects the interplay of multiple baseline and treatment-related factors rather than a single dominant determinant [3, 25]. Recent guideline updates, including ECIL-10, emphasize risk stratification by integrating both pre-infusion (e.g., prior therapies, baseline cytopenia, or prior IFI) and post-infusion factors (e.g., CRS requiring corticosteroids, prolonged neutropenia, or additional immunosuppressive therapy) [51]. The NCCN guidelines for MM further define high-risk scenarios based on treatment-related immunosuppression, including corticosteroid exposure (≥ 10 mg dexamethasone daily for > 3 days within a 7-day period), receipt of ≥ 1 g methylprednisolone per day, more than one dose of tocilizumab, or the use of additional immunosuppressive agents such as anakinra or siltuximab [32]. In this context, persistent respiratory symptoms or unexplained fever should prompt early imaging and evaluation of IFI [63, 85].

### CMV reactivation

CMV reactivation is increasingly recognized after CAR T-cell therapy, typically occurring within the first month after infusion, with a reported incidence ranging from approximately 17% to 56% and clinically significant infection in a smaller subset [86–89].

In contrast to allo-HSCT, current guidelines do not support universal CMV surveillance in CAR T-cell recipients [16, 56, 57]. The ECIL-10 recommendations suggest a risk-adapted approach, in which monitoring is considered primarily in CMV-seropositive patients with additional risk factors such as severe CRS, prolonged corticosteroid exposure, use of multiple immunosuppressive agents, or persistent lymphocytopenia. In these patients, CMV PCR monitoring may be performed during the early post-infusion period, typically between 2 and 6 weeks after CAR T-cell therapy, with the frequency adjusted according to the clinical course and immunosuppressive burden [26].

Preemptive therapy is generally initiated in the setting of high or rapidly increasing CMV DNAemia. However, ECIL-10 emphasizes that no universal viral load threshold can be defined and treatment decisions should be based on center-specific cut-offs, considering assay performance and calibration using WHO International Standard Units (IU/mL). From a clinical perspective, it is important to distinguish asymptomatic CMV DNAemia from clinically significant CMV infection and CMV end-organ disease because these entities have different management implications. When treatment is indicated, antiviral options, such as valganciclovir, ganciclovir, and foscarnet, may be considered. In the CAR T-cell setting, drug selection should account for toxicity profiles, particularly myelosuppression associated with ganciclovir-based therapies, which may limit their use in patients with ongoing cytopenia.

Given the absence of prospective CAR-T cell-specific data, current CMV monitoring and management strategies are largely extrapolated from the HSCT experience, which may limit their direct applicability in this setting.

## Conclusions

Current infection prevention strategies after CAR T-cell therapy are largely extrapolated from experience with intensive chemotherapy and HSCT and are primarily based on observed infection patterns rather than on evidence from randomized controlled trials. As CAR T-cell therapy continues to expand, more individualized approaches are needed. In this context, integrated patient-specific risk assessments may play an increasingly important role in guiding monitoring and preventive strategies. Biomarker-guided strategies that incorporate dynamic CD4^+^ T cell counts, B cell recovery, and immunoglobulin levels may help refine risk-adapted prevention, although prospective validation remains necessary. In addition, the infection risk appears to vary across CAR T constructs, particularly with a more profound and sustained humoral deficiency observed in BCMA-directed therapy. As next-generation cellular therapies, including newer targets and dual-target constructs, are introduced into clinical practice, defining their long-term infectious profiles will be an important priority for future research.

## Data Availability

No datasets were generated or analysed during the current study.

## References

[1] B-Cell Lymphomas. NCCN Clinical Practice Guidelines in Oncology. Version 1.2026.

[2] Sehn LH, Salles G. Diffuse large B-cell lymphoma. N Engl J Med. 2021;384(9):842–58. 33657296 10.1056/NEJMra2027612 PMC8377611

[3] Brudno JN, Maus MV, Hinrichs CS. CAR T cells and T-cell therapies for cancer: a translational science review. JAMA. 2024;332(22):1924–35. 39495525 10.1001/jama.2024.19462 PMC11808657

[4] Neelapu SS, et al. Comparison of 2-year outcomes with CAR T cells (ZUMA-1) vs salvage chemotherapy in refractory large B-cell lymphoma. Blood Adv. 2021;5(20):4149–55. 34478487 10.1182/bloodadvances.2020003848 PMC8945634

[5] A VS, et al. Tisagenlecleucel vs. historical standard of care in children and young adult patients with relapsed/refractory B-cell precursor acute lymphoblastic leukemia. Leukemia. 2023;37(12):2346–55. 37880478 10.1038/s41375-023-02042-4 PMC10681894

[6] Puig N, et al. Measurable residual disease by mass spectrometry and next-generation flow to assess treatment response in myeloma. Blood. 2024;144(23):2432–8. 39293025 10.1182/blood.2024024995

[7] Cappell KM, Kochenderfer JN. Long-term outcomes following CAR T cell therapy: what we know so far. Nat Rev Clin Oncol. 2023;20(6):359–71. 37055515 10.1038/s41571-023-00754-1 PMC10100620

[8] Bock TJ, et al. Outcome correlates of approved CD19-targeted CAR T cells for large B cell lymphoma. Nat Rev Clin Oncol. 2025;22(4):241–61. 39966627 10.1038/s41571-025-00992-5

[9] Kuhnl A, et al. A national service for delivering CD19 CAR-Tin large B-cell lymphoma - the UK real-world experience. Br J Haematol. 2022;198(3):492–502. 35485402 10.1111/bjh.18209

[10] Czapka MT, Riedell PA, Pisano JC. Infectious complications of CAR T-cell therapy: a longitudinal risk model. Transpl Infect Dis. 2023;25(Suppl 1):e14148. 37695203 10.1111/tid.14148

[11] Cordas Dos Santos DM, et al. A systematic review and meta-analysis of nonrelapse mortality after CAR T cell therapy. Nat Med. 2024;30(9):2667–78. 38977912 10.1038/s41591-024-03084-6 PMC11765209

[12] Lemoine J, et al. Nonrelapse mortality after CAR T-cell therapy for large B-cell lymphoma: a LYSA study from the DESCAR-T registry. Blood Adv. 2023;7(21):6589–98. 37672383 10.1182/bloodadvances.2023010624 PMC10641092

[13] Penack O, et al. Organ complications after CD19 CAR T-cell therapy for large B cell lymphoma: a retrospective study from the EBMT transplant complications and lymphoma working party. Front Immunol. 2023;14:1252811. 37828980 10.3389/fimmu.2023.1252811 PMC10565347

[14] Kampouri E, et al. Infections after chimeric antigen receptor (CAR)-T-cell therapy for hematologic malignancies. Transpl Infect Dis. 2023;25(Suppl 1):e14157. 37787373 10.1111/tid.14157

[15] Haidar G, Garner W, Hill JA. Infections after anti-CD19 chimeric antigen receptor T-cell therapy for hematologic malignancies: timeline, prevention, and uncertainties. Curr Opin Infect Dis. 2020;33(6):449–57. 33009139 10.1097/QCO.0000000000000679

[16] Prevention and Treatment of Cancer-Related Infections*.* NCCN(National Comprehensive Cancer Network). Version 2.2025-January 22, 2026.

[17] Management of CAR T-Cell and Lymphocyte Engager-Related Toxicities*.* NCCN Clinical Practice Guidelines in Oncology. Version 2.2026-November 11, 2025.

[18] Telli Dizman G, Aguado JM, Fernandez-Ruiz M. Risk of infection in patients with hematological malignancies receiving CAR T-cell therapy: systematic review and meta-analysis. Expert Rev Anti Infect Ther. 2022;20(11):1455–76. 36148506 10.1080/14787210.2022.2128762

[19] Wudhikarn K, et al. Infection after CD19 chimeric antigen receptor T-cell therapy for large B-cell lymphoma: real-world analysis from CIBMTR. Blood Adv. 2025;9(21):5460–72. 40435511 10.1182/bloodadvances.2025016141 PMC12607006

[20] Jain MD, Smith M, Shah NN. How I treat refractory CRS and ICANS after CAR T-cell therapy. Blood. 2023;141(20):2430–42. 36989488 10.1182/blood.2022017414 PMC10329191

[21] Rejeski K, et al. CAR-HEMATOTOX: a model for CAR T-cell-related hematologic toxicity in relapsed/refractory large B-cell lymphoma. Blood. 2021;138(24):2499–513. 34166502 10.1182/blood.2020010543 PMC8893508

[22] Rejeski K, Subklewe M, Locke FL. Recognizing, defining, and managing CAR-T hematologic toxicities. Hematology Am Soc Hematol Educ Program. 2023;2023(1):198–208. 38066881 10.1182/hematology.2023000472 PMC10727074

[23] Rejeski K, et al. Immune effector cell-associated hematotoxicity: EHA/EBMT consensus grading and best practice recommendations. Blood. 2023;142(10):865–77. 37300386 10.1182/blood.2023020578

[24] Si X, et al. Hematologic cytopenia post CAR T cell therapy: etiology, potential mechanisms and perspective. Cancer Lett. 2022;550:215920. 36122628 10.1016/j.canlet.2022.215920

[25] Bouvier A, et al. Invasive fungal infections after CD19 chimeric antigen receptor T-cell therapy for B-cell lymphoma: a Lymphoma study association study from the DESCAR-T (Dispositif d’Enregistrement et Suivi des patients traites par CAR-T cells) registry. Clin Microbiol Infect. 2026;32(2):277–84. 41109429 10.1016/j.cmi.2025.10.005

[26] Ljungman P, et al. Recommendations from the 10th European Conference on Infections in Leukaemia for the management of cytomegalovirus in patients after allogeneic haematopoietic cell transplantation and other T-cell-engaging therapies. Lancet Infect Dis. 2025;25(8):e451–62. 40188837 10.1016/S1473-3099(25)00069-6

[27] Sassine J, Siegrist EA, Chemaly RF. Herpesvirus infections after Chimeric Antigen Receptor T-Cell Therapy and bispecific antibodies: a review. Viruses. 2025. 10.3390/v17010133. 39861922 10.3390/v17010133 PMC11768728

[28] Hematopoietic Growth Factors*.* NCCN Clinical Practice Guidelines in Oncology. Version 3.2026.

[29] Nakamura N, et al. Clinical impact of cytokine release syndrome on prolonged hematotoxicity after chimeric antigen receptor T Cell therapy: KyoTox A-Score, a novel prediction model. Transplant Cell Ther. 2024;30(4):404–14. 38281589 10.1016/j.jtct.2024.01.073

[30] Bishop MR. Late complications and long-term care of adult CAR T-cell patients. Hematology Am Soc Hematol Educ Program. 2024;2024(1):109–15. 39643985 10.1182/hematology.2024000534 PMC11665735

[31] Kampouri E, et al. Managing hypogammaglobulinemia in patients treated with CAR-T-cell therapy: key points for clinicians. Expert Rev Hematol. 2022;15(4):305–20. 35385358 10.1080/17474086.2022.2063833

[32] Multiple Myeloma. NCCN Clinical Practice Guidelines in Oncology. Version 5.2026-January 9.2026. 10.6004/jnccn.2026.0001 41671464

[33] Santomasso BD, et al. Management of immune-related adverse events in patients treated with chimeric antigen receptor T-cell therapy: ASCO guideline. J Clin Oncol. 2021;39(35):3978–92. 34724386 10.1200/JCO.21.01992

[34] Riedel A, et al. Immunological consequences of CAR T-cell therapy: an analysis of infectious complications and immune reconstitution. Blood Adv. 2025;9(13):3149–58. 40188456 10.1182/bloodadvances.2024015346 PMC12242403

[35] Reynolds GK, et al. Infections in haematology patients treated with CAR-T therapies: a systematic review and meta-analysis. Crit Rev Oncol Hematol. 2023;192:104134. 37739146 10.1016/j.critrevonc.2023.104134

[36] Hill JA, Seo SK. How I prevent infections in patients receiving CD19-targeted chimeric antigen receptor T cells for B-cell malignancies. Blood. 2020;136(8):925–35. 32582924 10.1182/blood.2019004000 PMC7441168

[37] van de Donk N, et al. Optimising T-cell immunotherapy in patients with multiple myeloma: practical considerations from the European Myeloma Network. Lancet Haematol. 2025;12(8):e635–49. 40580975 10.1016/S2352-3026(25)00117-6

[38] Wang Y, et al. Humoral immune reconstitution after anti-BCMA CAR T-cell therapy in relapsed/refractory multiple myeloma. Blood Adv. 2021;5(23):5290–9. 34587230 10.1182/bloodadvances.2021004603 PMC9153033

[39] van de Donk N, Usmani SZ, Yong K. CAR T-cell therapy for multiple myeloma: state of the art and prospects. Lancet Haematol. 2021;8(6):e446–61. 34048683 10.1016/S2352-3026(21)00057-0

[40] Josyula S, et al. Pathogen-Specific Humoral Immunity and Infections in B Cell Maturation Antigen-Directed Chimeric Antigen Receptor T Cell Therapy Recipients with Multiple Myeloma. Transplant Cell Ther, 2022;28(6):304. e1–304 e9. 10.1016/j.jtct.2022.03.005 PMC9197988 35288345

[41] Wat J, Barmettler S. Hypogammaglobulinemia after chimeric antigen receptor (CAR) T-cell therapy: characteristics, management, and future directions. J Allergy Clin Immunol Pract. 2022;10(2):460–6. 34757064 10.1016/j.jaip.2021.10.037 PMC8837681

[42] Maziarz RT, et al. Five-year analysis of the JULIET trial of Tisagenlecleucel in patients with relapsed/refractory large B-Cell lymphoma. J Clin Oncol. 2026;44(2):86–91. 41252666 10.1200/JCO-25-00507 PMC12783362

[43] Neelapu SS, et al. Five-year follow-up of ZUMA-1 supports the curative potential of axicabtagene ciloleucel in refractory large B-cell lymphoma. Blood. 2023;141(19):2307–15. 36821768 10.1182/blood.2022018893 PMC10646788

[44] Yang Y, et al. New insights into CAR T-cell hematological toxicities: manifestations, mechanisms, and effective management strategies. Exp Hematol Oncol. 2024;13(1):110. 39521987 10.1186/s40164-024-00573-9 PMC11549815

[45] Park JH, et al. Cytokine release syndrome grade as a predictive marker for infections in patients with relapsed or refractory B-Cell acute lymphoblastic leukemia treated with chimeric antigen receptor T Cells. Clin Infect Dis. 2018;67(4):533–40. 29481659 10.1093/cid/ciy152 PMC6070095

[46] Brockelmann PJ, et al. Beyond maximum grade: tolerability of immunotherapies, cellular therapies, and targeted agents in haematological malignancies. Lancet Haematol. 2025;12(6):e470–81. 40447355 10.1016/S2352-3026(25)00051-1

[47] Arya S, Shahid Z. Overview of infectious complications among CAR T- cell therapy recipients. Front Oncol. 2024;14:1398078. 39026972 10.3389/fonc.2024.1398078 PMC11255439

[48] Wang M, et al. KTE-X19 CAR T-Cell therapy in relapsed or refractory mantle-cell lymphoma. N Engl J Med. 2020;382(14):1331–42. 32242358 10.1056/NEJMoa1914347 PMC7731441

[49] Jain T, Olson TS, Locke FL. How I treat cytopenias after CAR T-cell therapy. Blood. 2023;141(20):2460–9. 36800563 10.1182/blood.2022017415 PMC10646792

[50] Rejeski K, et al. Immune effector cell-associated haematotoxicity after CAR T-cell therapy: from mechanism to management. Lancet Haematol. 2024;11(6):e459–70. 38734026 10.1016/S2352-3026(24)00077-2 PMC12413773

[51] Pagano L, et al. Primary antifungal prophylaxis in hematological malignancies. Updated clinical practice guidelines by the European Conference on Infections in Leukemia (ECIL). Leukemia. 2025;39(7):1547–57. 40200079 10.1038/s41375-025-02586-7 PMC12208874

[52] Lievin R, et al. Effect of early granulocyte-colony-stimulating factor administration in the prevention of febrile neutropenia and impact on toxicity and efficacy of anti-CD19 CAR-T in patients with relapsed/refractory B-cell lymphoma. Bone Marrow Transplant. 2022;57(3):431–9. 35094012 10.1038/s41409-021-01526-0 PMC8907072

[53] Schubert ML, et al. Side-effect management of chimeric antigen receptor (CAR) T-cell therapy. Ann Oncol. 2021;32(1):34–48. 33098993 10.1016/j.annonc.2020.10.478

[54] Gudiol C, et al. Chimeric antigen receptor T-cell therapy for the treatment of lymphoid malignancies: is there an excess risk for infection? Lancet Haematol. 2021;8(3):e216–28. 33460558 10.1016/S2352-3026(20)30376-8

[55] Stemler J, et al. Primary prophylaxis of invasive fungal diseases in patients with haematological malignancies: 2022 update of the recommendations of the Infectious Diseases Working Party (AGIHO) of the German Society for Haematology and Medical Oncology (DGHO). J Antimicrob Chemother. 2023;78(8):1813–26. 37311136 10.1093/jac/dkad143 PMC10393896

[56] Greco R, et al. Indications for haematopoietic cell transplantation and CAR-T for haematological diseases, solid tumours and immune disorders: 2025 EBMT practice recommendations. Bone Marrow Transplant. 2025;60(11):1499–525. 40926035 10.1038/s41409-025-02701-3 PMC12583170

[57] Shahid Z, et al. Best practice considerations by The American Society of Transplant and Cellular Therapy: infection prevention and management after chimeric antigen receptor T cell therapy for hematological malignancies. Transplant Cell Ther. 2024;30(10):955–69. 39084261 10.1016/j.jtct.2024.07.018 PMC12826105

[58] Taplitz RA, et al. Antimicrobial prophylaxis for adult patients with cancer-related immunosuppression: ASCO and IDSA clinical practice guideline update. J Clin Oncol. 2018;36(30):3043–54. 30179565 10.1200/JCO.18.00374

[59] Liu C, et al. Tackling antimicrobial resistance in people who are immunocompromised: leveraging diagnostic and antimicrobial stewardship. Lancet Infect Dis. 2026;26(1):e30–48. 40812337 10.1016/S1473-3099(25)00311-1 PMC13218428

[60] Al-Ramahi JS, et al. Lessons learned from COVID-19 pandemic: outcomes after SARS-CoV-2 infection in hematopoietic cell transplant and cell therapy recipients. Leuk Lymphoma. 2023;64(12):1981–91. 37574842 10.1080/10428194.2023.2243355

[61] Rosen EA, et al. COVID-19 outcomes among hematopoietic cell transplant and chimeric antigen receptor T-cell recipients in the era of SARS-CoV-2 Omicron variants and COVID-19 therapeutics. Transplant Cell Ther. 2024;30(11):1108 e1-1108 e11. 39179107 10.1016/j.jtct.2024.08.010 PMC11540736

[62] Tabbara N, Dioverti-Prono MV, Jain T. Mitigating and managing infection risk in adults treated with CAR T-cell therapy. Hematology Am Soc Hematol Educ Program. 2024;2024(1):116–25. 39644015 10.1182/hematology.2024000535 PMC11706248

[63] Pennese E, et al. Case report: invasive fungal infection after anti-CD19 CAR-T cell therapy. Implication for antifungal prophylaxis. Front Immunol. 2023;14:1272798. 37841271 10.3389/fimmu.2023.1272798 PMC10574963

[64] Dadwal SS, et al. American Society of Transplantation and Cellular Therapy Series, 2: management and prevention of aspergillosis in hematopoietic cell transplantation recipients. Transplant Cell Ther. 2021;27(3):201–11. 33781516 10.1016/j.jtct.2020.10.003 PMC9088165

[65] Kamboj M, et al. Vaccination of adults with cancer: ASCO guideline. J Clin Oncol. 2024;42(14):1699–721. 38498792 10.1200/JCO.24.00032 PMC11095883

[66] Survivorship. NCCN Clinical Practice Guidelines in Oncology. Version 3.2025 - February 2, 2026.

[67] Einarsdottir S, et al. Humoral vaccine responses following chimeric antigen receptor T-cell therapy for hematological malignancies. Blood Cancer J. 2025;15(1):114. 40603286 10.1038/s41408-025-01321-w PMC12223166

[68] Aleissa MM, et al. Severe Acute Respiratory Syndrome Coronavirus 2 Vaccine Immunogenicity among Chimeric Antigen Receptor T Cell Therapy Recipients. Transplant Cell Ther. 2023;29(6):398. e1–398 e5. 10.1016/j.jtct.2023.03.005 PMC9995387 36906276

[69] Lee D, et al.Pneumococcal Conjugate Vaccine Does Not Induce Humoral Response When Administrated Within the Six Months After CD19 CAR T-Cell Therapy. Transplant Cell Ther. 2023;29(4):277. e1–277 e9. 10.1016/j.jtct.2022.08.011 35970303

[70] Khawaja F, et al. Frequently asked questions on Coronavirus Disease 2019 vaccination for hematopoietic cell transplantation and chimeric antigen receptor T-Cell recipients from the American Society for Transplantation and Cellular Therapy and the American Society of Hematology. Transplant Cell Ther. 2023;29(1):10–8. 36273782 10.1016/j.jtct.2022.10.010 PMC9584756

[71] Hill JA, et al. SARS-CoV-2 vaccination in the first year after hematopoietic cell transplant or chimeric antigen receptor T-Cell therapy: A prospective, multicenter, observational study. Clin Infect Dis. 2024;79(2):542–54. 38801746 10.1093/cid/ciae291 PMC11327798

[72] Gossi S, et al. Humoral responses to repetitive doses of COVID-19 mRNA vaccines in patients with CAR-T-Cell Therapy. Cancers (Basel). 2022. 10.3390/cancers14143527. 35884587 10.3390/cancers14143527 PMC9319387

[73] Kinoshita H, et al. T Cell immune response to Influenza Vaccination when administered prior to and following autologous Chimeric Antigen Receptor-Modified T Cell Therapy. Transplant Cell Ther. 2025;31(5):327–38. 40032074 10.1016/j.jtct.2025.02.019 PMC12058394

[74] Pediatric Acute Lymphoblastic Leukemia. NCCN Clinical Practice Guidelines in Oncology. Version 1.2026.

[75] Otani IM, et al. Practical guidance for the diagnosis and management of secondary hypogammaglobulinemia: a work group report of the AAAAI Primary Immunodeficiency and Altered Immune Response Committees. J Allergy Clin Immunol. 2022;149(5):1525–60. 35176351 10.1016/j.jaci.2022.01.025

[76] Wonnaparhown A, et al. IgG replacement in multiple myeloma. Blood Cancer J. 2024;14(1):124. 39054331 10.1038/s41408-024-01107-6 PMC11272770

[77] Ludwig H, et al. Prevention and management of adverse events during treatment with bispecific antibodies and CAR T cells in multiple myeloma: a consensus report of the European Myeloma Network. Lancet Oncol. 2023;24(6):e255–69. 37269857 10.1016/S1470-2045(23)00159-6

[78] Ali FS, et al. AGA Clinical Practice Guideline on the prevention and treatment of Hepatitis B Virus reactivation in at-risk individuals. Gastroenterology. 2025;168(2):267–84. 39863345 10.1053/j.gastro.2024.11.008

[79] Mikulska M, et al. Updated recommendations for the management of hepatitis B, C, and E virus infections in patients with haematological malignancies and those undergoing haematopoietic cell transplantation: recommendations from the 9th European Conference on Infections in Leukaemia (ECIL-9). Lancet Haematol. 2025;12(5):e389–99. 40306834 10.1016/S2352-3026(25)00049-3

[80] Los-Arcos I, et al. Recommendations for screening, monitoring, prevention, and prophylaxis of infections in adult and pediatric patients receiving CAR T-cell therapy: a position paper. Infection. 2021;49(2):215–31. 32979154 10.1007/s15010-020-01521-5 PMC7518951

[81] Infante MS, et al. Outcomes and Management of the SARS-CoV2 Omicron Variant in Recipients of Hematopoietic Cell Transplantation and Chimeric Antigen Receptor T Cell Therapy. Transplant Cell Ther. 2024;30(1):116. e1–116 e12. 10.1016/j.jtct.2023.09.027 PMC11220618 37806446

[82] Mushtaq MU, et al. Impact of SARS-CoV-2 in Hematopoietic Stem Cell Transplantation and Chimeric Antigen Receptor T Cell Therapy Recipients. Transplant Cell Ther 2021;27(9):796. e1–796 e7. 10.1016/j.jtct.2021.07.005 PMC8272625 34256172

[83] Luque-Paz D, et al. The burden of SARS-CoV-2 in patients receiving chimeric antigen receptor T cell immunotherapy: everything to lose. Expert Rev Anti Infect Ther. 2022;20(9):1155–62. 35838042 10.1080/14787210.2022.2101448

[84] Kampouri E, Hill JA, Dioverti V. COVID-19 after hematopoietic cell transplantation and chimeric antigen receptor (CAR)-T-cell therapy. Transpl Infect Dis. 2023;25(Suppl 1):e14144. 37767643 10.1111/tid.14144

[85] Cheok KPL, et al. Mucormycosis after CD19 chimeric antigen receptor T-cell therapy: results of a US Food and Drug Administration adverse events reporting system analysis and a review of the literature. Lancet Infect Dis. 2024;24(4):e256–65. 38310904 10.1016/S1473-3099(23)00563-7

[86] Lin RY, et al. Incidence and outcomes of Cytomegalovirus reactivation after chimeric antigen receptor T-cell therapy. Blood Adv. 2024;8(14):3813–22. 38838226 10.1182/bloodadvances.2024012922 PMC11298821

[87] Marquez-Algaba E, et al. Impact of cytomegalovirus replication in patients with aggressive B cell lymphoma treated with chimeric antigen receptor T cell therapy. Transplant Cell Ther. 2022;28(12):851 e1-851 e8. 36221995 10.1016/j.jtct.2022.09.007

[88] Hayashino K, et al. Cytomegalovirus reactivation in patients with large B-cell lymphoma treated with chimeric antigen receptor T-cell therapy. Int J Hematol. 2025;122(5):689–99. 40526215 10.1007/s12185-025-04023-y PMC12572091

[89] Khan MA, et al. Predicators and outcomes of Cytomegalovirus reactivation in chimeric antigen receptor T-cell therapy: A systematic review. Clin Lymphoma Myeloma Leuk. 2025;25(12):e1121–6. 40940256 10.1016/j.clml.2025.08.014

